# Attitudes towards urban stray cats and managing their population in India: a pilot study

**DOI:** 10.3389/fvets.2023.1274243

**Published:** 2023-10-27

**Authors:** Anamika Changrani-Rastogi, Nishakar Thakur

**Affiliations:** ^1^Dhirubhai Ambani International School, Mumbai, India; ^2^All India Institute of Medical Sciences, New Delhi, India

**Keywords:** animal welfare, urban animals, trap-neuter-return, feline, population control, attitudes

## Abstract

Life in contemporary cities is often dangerous for stray cats, with strikingly low survival rates. In several countries, trap-neuter-return (TNR) programs have been employed to control urban stray cat populations. Management of stray cats in urban environments is not just about applying scientific solutions, but also identifying approaches that align with local cultural and ethical values. India has an estimated 9.1 million stray cats. TNR presents as a potential method for stray cat management in India, while also improving their welfare. Yet, to date, there has been no academic exploration on Indian residents’ attitudes towards stray cats. We conducted a survey in 13 cities in India reaching 763 residents, examining interactions with stray cats, negative and positive attitudes towards them, attitudes towards managing their population, and awareness of TNR. Results show a high rate of stray cat sightings and interactions. While most respondents believed that stray cats had a right to welfare, the majority held negative attitudes towards and had negative interactions with them. There was widespread lack of awareness about TNR, but, when described, there was a high degree of support. Gathering insights into opinions about stray cats, and the sociodemographic factors that impact these opinions, is an important first step to developing policies and initiatives to manage stray cat populations.

## 1. Introduction

Unowned urban stray cats present important challenges across the world (1). The safety of urban stray cats is often threatened, with an estimated mortality rate of 75–90% for kittens by 6 months of age (2). Welfare concerns also include starvation, abuse, and disease (3). In many countries, colonies of urban stray cats are viewed as a problem to be solved by humans (4). Problems cited include disease transmission to humans, disturbing noises, and excrement and odors (5). For those with the cats’ welfare in mind, the concern regarding the high mortality in stray cats is viewed as problematic (6). As such, it is accepted as essential to control urban stray cat populations, both for their welfare and that of human residents.

Popular strategies for controlling stray cat populations include trap-neuter-return (TNR), mass euthanasia, and removal for adoption (7). Of these, TNR is regarded as most humane, and is popular in the wake of a growing “no-kill movement” (8). The primary objectives of a TNR program are stabilization and reduction of stray cat numbers (9). The essential elements of a TNR program include humane trapping, surgically rendering cats sterile, ear-tip-cutting for identification, rabies vaccination and returning the cat to the original territory (9). In urban areas in particular, TNR has been successful in controlling cat colonies (10). In several countries, TNR programs have been effectively deployed to managing urban cat populations (10–14). Several studies, including longitudinal studies, have shown success not only in population control but also reducing complaints of nuisance such as odor and noise (11, 13, 15–17).

To date, research into managing urban stray cat populations has largely been conducted in high income and upper middle income countries such as Australia, Belgium, Bulgaria, Israel, Italy, Japan, New Zealand, and the United States (1, 10–15, 18, 19). Low and lower-middle income countries such as India face considerable challenges with urban stray cats. India has witnessed persistent population growth in urban areas, and globally, Indian urban agglomerations are second only to China (20). An overlooked concern with massive urban growth and its attendant challenges is the welfare of stray cats (21). India had an estimated 9.1 million stray cats in 2021 (22). In 2018, the Animal Welfare Board of India (AWBI) issued an advisory for State Governments, noting the problem of overpopulation of cats in urban areas (23). The AWBI detailed the plight of stray cats, including suffering from trauma, exposure, accidents and starvation. It also observed that kicking and hitting stray cats was not uncommon (23).

There is a need for research on attitudes towards urban stray cats in low and lower-middle income countries, alongside efforts examining approaches to controlling their population. In order for TNR to be effective, community engagement is essential (24). Studies show attitudes towards managing stray cat populations are shaped by a multitude of factors including owning a cat (25, 26), place of residence (26–29), gender (30), age (31), education (32), and religion (33) among others. Further, public education campaigns accompanying efforts to manage stray cat populations need to be tailored to the audience based on attitudes towards and beliefs about stray cats and population control (8, 34). Consequently, understanding local attitudes towards stray cats and approaches to controlling their population is important for the success of policies and interventions to address overpopulation of urban stray cats.

To date, there has been no study exploring opinions on stray cats in India. Our study sought to understand attitudes towards, and perceptions of, stray cats among urban residents across India. We also explored attitudes towards stray cat population control. As such, our study is the first step in gathering information to guide strategies in stray cat population control in India.

## 2. Methods

### 2.1. Terminology used

Various terms are used to describe cats living alongside humans including unowned cats, free-ranging cats, stray cats, community cats, or feral cats (35, 36). We used “stray” cats as is commonly understood in India. Further, the common phrase in India for TNR is sterilization, used in programs to control the stray dog population. Accordingly, we used the term “sterilization” in our survey.

### 2.2. Survey development

A 29-item questionnaire was developed. A literature review was conducted on determining attitudes towards animals in general, cats and stray cats in particular, and towards population control, including questions on TNR (26, 29, 34, 37, 38). Databases used included MEDLINE, PubMed and GoogleScholar. Search terms for describing stray cats included community, feral, free-ranging, stray, and unowned cats. Eligible articles included those reporting qualitative or quantitative original research and published in English. No restrictions were placed on publication dates.

Questionnaires on feeding cats were reviewed (38, 39). The draft questionnaire was reviewed by four cat-welfare international experts, and one researcher on animal behavior and ecology in India, and edited based on their feedback. A pilot questionnaire in English was administered to 20 residents in three Mumbai neighborhoods, leading to one question being removed and phrasing edits in two questions. The final questionnaire included 29 questions, including 10 questions on a 5-point Likert-response scale (Strongly Agree, Somewhat Agree, Unsure, Somewhat Disagree, Strongly Disagree), 8 questions with Yes/No options, and 11 questions on pet ownership and demographics.

The questions explored:

- Sighting and interactions with stray cats, including whether respondents have seen a stray cat where they live/work/study, whether seen in the past month, whether they have shooed off a stray cat, been bitten/scratched by a stray cat, fed them, taken any to the vet, and if they have adopted any cats off the street.
- Attitudes towards stray cats, both negative and positive, whether the welfare of stray cats is important, whether they believe stray cats are dirty, stinking up the place where they are, whether they are a nuisance to humans causing disturbance with loud fighting. Beliefs on whether stray cats spread diseases to humans was explored. One question asked whether people who feed stray cats are creating a bigger problem, while another asked whether feeding a stray cat would make them feel good.
- Attitudes towards the management of stray cat population and awareness of TNR. Attitude questions included whether they supported euthanasia, referencing the now-illegal approach to reducing stray dog numbers in India, whether they supported sterilizing (neutering), and whether local governments/municipal corporations should have sterilizing (TNR) programs.

The questionnaire was translated into seven regional Indian languages including Bengali, Gujarati, Hindi, Kannada, Malayam, Marathi and Tamil. The English version of the questionnaire was made available online using Google Forms.

### 2.3. Data collection

A data collection firm with offices and full-time staff interviewers in 13 cities across India was engaged. 19 interviewers were trained and overseen by a project manager. Interviewers were provided with gender/age quotas. The survey was administered in public locations by interviewers using quota convenience sampling. Potential participants were presented with a brief oral scripted study description. They were informed that no identifying information would be collected and their data would remain anonymous. Once participants provided consent, the interviewer proceeded to ask questions orally in their preferred language, while selecting responses in the English survey version online on a tablet.

### 2.4. Statistical analysis

The data underwent outlier screening, followed by descriptive analysis utilizing frequency counts to assess attitudes towards stray cats, interactions with stray cats, pet/cat ownership, and demographic variables. To investigate potential influencing factors on attitudes towards stray cats, their population management, and interactions with stray cats, measures of association between these variables were examined using the Chi-square test statistic. Variables with *p*-values <0.05 were deemed statistically significant. The following characteristics were chosen as independent variables: gender, age (young <30 years versus old ≥30 years), education level (some college/graduation from college versus no college), pet-ownership, and cat-ownership. Statistical analysis was done using STATA 16 (40).

## 3. Results

A total of 763 surveys were completed across 13 cities including Ahmedabad (9.17%), Bengaluru (9.44%), Bhubaneshwar (4.06%), Chennai (11.66%), Delhi (6.95%), Kochi (10.62%), Kolkata (8.39%), Lucknow (10.77%), Mumbai (13.63%), Nagpur (3.93%), Patna (6.55%), Varanasi (3.28%), and Vijayawada (2.23%). Only 0.3% of survey items were left unanswered. 47% of participants were female, 52.7% male, 0.3% “would rather not say.” 37.2% were < 30 years old, 35.7% 30–44 years, and the rest ≥45 years. 63.3% had completed some/all college-level education. 36.4% reported ever owning a pet, including 22.7% reporting cats.

738 respondents (96.7%) said they were aware of stray cats in their neighborhood where they lived/worked/studied, and all but one of them had seen a stray cat in the past month.

37.6% of respondents had at some point shooed off a stray cat who they thought was being a nuisance, 61.3% reported never shooing off a stray cat and 1.1% unanswered. Females and older respondents were more likely to report that they had shooed off a cat, as had respondents who never had pets.

18.6% of respondents had at some point been bitten/scratched by a stray cat, whereas 80.9% had not (0.5% missing). Those who feed stray cats were more likely to have been scratched/bitten by them. However, 9% of the respondents who had never fed any stray cats reported being bitten/scratched.

36.7% of respondents reported ever feeding a stray cat (Table 1). Cat feeders were across both genders, young and old, with and without college education in similar proportions with no statistically significant differences.

**Table 1:** Survey items regarding supporting stray cats’ health/welfare: response distributions and chi-square differences in response distributions by demographic variables.

Survey item (number of valid responses for item)	Response proportions (as % and frequencies)	Demographic variables tested (number of respondents)	Pearson’s chi-square statistic and degrees of freedom	*p*-values
Do you feed or have you ever fed any stray cats?	Yes = 280, 36.70%	Gender	1.16	0.5592
	No = 483, 63.30%	Age	1.88	0.5977
		Education	12.41	0.0146
		Own-pet	244.85	0.0000
Have you ever taken a stray cat to a vet?	Yes = 110, 14.42%	Gender	0.74	0.6919
	No = 650, 85.19%	Age	11.68	0.0086
		Education	16.18	0.0028
		Own-pet	240.39	0.0000
Have you ever adopted a stray cat and taken it home?	Yes = 116, 15.20%	Gender	3.88	0.1437
	No = 647, 84.80%	Age	9.53	0.0230
		Education	14.25	0.0065

14.4% of respondents reported ever taking a stray cat to a vet. Young people <30 years were more likely to have taken a stray cat to a vet (18.5%) compared with ≥30 years (12.1%) (Pearson Chi2 = 5.85 Prob = 0.016). No significant difference was noted in taking a stray cat to a vet by gender (Females 15.4%, Males 13.8%, *p* = 0.7), education (Some college/ more 15.4%, No college 12.9%, *p* = 0.33).

15.2% of respondents reported adopting a stray cat. Adopters were across both genders, young and old ages, with or without college education, with no statistically significant difference.

### 3.1. Perceived nuisance behaviors of stray cats

More participants agreed (somewhat agreed/strongly agreed) than disagreed (somewhat disagreed/strongly disagreed) that stray cats are dirty and stink up the place where they are, and are a nuisance and disturb humans with their loud fighting (Table 2). Many respondents did not hold an opinion and were unsure about these items regarding perceived nuisance. Older respondents and those with some college education were more inclined to agree with the nuisance behavior items. Pet-owners and cat-owners were more likely to not perceive nuisance behaviors and disagreed (somewhat/strongly) with both nuisance behavior items. No significant difference across genders was noted.

**Table 2:** Survey items regarding nuisance related to stray cats: response distributions and chi-square differences in response distributions by demographic variables.

Survey item (number of valid responses for item)	Response proportions (as % and frequencies)	Demographic variables tested (number of respondents)	Pearson’s chi-square statistic and degrees of freedom	*p*-values
Stray cats are dirty and stink up the place where they are	SD = 71, 9.31%	Gender	9.73	0.2846
	D = 120, 15.73%	Age	19.83	0.0005
	N = 83, 10.88%	Education	10.34	0.0350
	A = 260, 34.08%	Own-pet	77.29	0.0000
	SA = 228, 29.88%			
Stray cats spread diseases to humans	SD = 69, 9.04%	Gender	10.77	0.2151
	D = 112, 14.68%	Age	16.36	0.0026
	N = 107, 14.02%	Education	5.02	0.2848
	A = 267, 34.99%	Own-pet	89.15	0.0000
	SA = 207, 27.13%			
Stray cats are a nuisance and disturb humans with their loud fighting	SD = 57, 7.47%	Gender	6.51	0.5906
	D = 163, 21.36%	Age	14.16	0.0068
	N = 79, 10.35%	Education	3.49	0.4799
	A = 267, 34.99%	Own-pet	92.71	0.0000
	SA = 197, 25.82%			
People who feed stray cats are creating a bigger problem	SD = 116, 15.20%	Gender	16.73	0.0330
	D = 192, 25.16%	Age	16.58	0.0023
	N = 158, 20.71%	Education	9.73	0.0452
	A = 154, 20.18%	Own-pet	110.66	0.0000
	SA = 142, 18.61%			

### 3.2. Perceived spread of disease from stray cats to humans

More participants agreed (somewhat agreed/strongly agreed) than disagreed (somewhat disagreed/strongly disagreed) that stray cats spread diseases to humans (Table 2). Older respondents were likelier to believe that stray cats spread diseases to humans. Pet-owners and cat-owners disagreed more than respondents with no pets. No significant difference in gender/education was noted for the perceived spread of disease.

### 3.3. Perceptions regarding feeding stray cats

When asked whether people who are feeding stray cats are creating a bigger problem, responses were mixed: 38.8% agreeing (strongly/somewhat), 40.4% disagreeing (strongly/somewhat), 20.7% unsure (Table 3). Older respondents and those without college education were more likely to agree that people who were feeding cats were creating a bigger problem. Pet-owners and cat-owners were more inclined to disagree. Males were more likely to say they were unsure, whereas females were more likely to agree.

**Table 3:** Survey items regarding welfare of stray cats: response distributions and chi-square differences in response distributions by demographic variables.

Survey item (number of valid responses for item)	Response proportions (as % and frequencies)	Demographic variables tested (number of respondents)	Pearson’s chi-square statistic and degrees of freedom	*p*-values
The welfare of stray cats is important	SD = 80, 10.48%	Gender	12.45	0.1323
	D = 110, 14.42%	Age	25.63	0.0000
	N = 97, 12.71%	Education	19.85	0.0005
	A = 214, 28.08%	Own-pet	111.92	0.0000
	SA = 260, 34.08%			
Feeding a stray cat would make me feel good	SD = 127, 16.64%	Gender	8.37	0.3986
	D = 142, 18.61%	Age	19.50	0.0006
	N = 128, 16.78%	Education	8.03	0.0905
	A = 174, 22.80%	Own-pet	169.37	0.0000
	SA = 188, 24.64%			
People who feed stray cats are improving the welfare of cats	SD = 75, 9.83%	Gender	8.23	0.4114
	D = 110, 14.42%	Age	15.99	0.0030
	N = 206, 27.00%	Education	10.44	0.0336
	A = 215, 28.81%	Own-pet	124.88	0.0000
	SA = 156, 20.45%			

### 3.4. Caring and humane attitudes towards stray cats

62.1% agreed (strongly/somewhat) with the statement “The welfare of stray cats is important,” 24.9% disagreed (strongly/somewhat), 12.7% were unsure (Table 3). Younger respondents, those with college education, pet-owners and cat owners were more likely to agree upon the importance of the welfare of stray cats. There was no significant difference across the genders.

In response to the item “feeding a stray cat would make me feel good,” 47.4% strongly/somewhat agreed, 35.3% disagreed, 16.8% were unsure (Table 3). Older respondents and those without college education expressed more disagreement, pet-owners and cat-owners expressed more agreement, and there was no significant difference across the genders.

### 3.5. Managing stray cat population

72.2% had never heard of TNR programs. Older respondents were more likely to have heard about TNR programs, as had respondents who had pets. There was no significant association with gender or education. Three items referred to the management of the stray cat population. For the first, “Local governments/municipal corporations should remove all the stray cats from the streets and euthanise (kill) them like they used to do with dogs” 56.6% disagreed (strongly/somewhat), 25.6% agreed (strongly/somewhat) and 17.8% were unsure (Table 4). Younger respondents were more likely to disagree, but there was no significant difference across genders/education-level.

**Table 4:** Survey items regarding population control of stray cats: response distributions and chi-square differences in response distributions by demographic variables and feeding history.

Survey item (number of valid responses for item)	Response proportions (as % and frequencies)	Demographic variables tested (number of respondents)	Pearson’s chi-square statistic and degrees of freedom	*p*-values
Stray cat numbers should be reduced by sterilizing them so they are unable to have more kittens	SD = 54, 17.08%	Gender	15.43	0.0514
	D = 107, 14.02%	Age	6.54	0.8867
	N = 106, 13.89%	Education	52.58	0.0000
	A = 260, 34.08%	Own-pet	110.11	0.0000
	SA = 235, 30.8%	Feeder (y/n)	85.02	0.0000
Local governments/municipal corporations should remove all the stray cats from the streets and euthanize (kill) them like they used to do with dogs	SD = 284, 37.22%	Gender	13.58	0.0935
	D = 148, 19.40%	Age	16.62	0.1645
	N = 136, 17.82%	Education	52.42	0.0000
	A = 121, 14.68%	Own-pet	164.86	0.0000
	SA = 83, 10.88%	Feeder (y/n)	160.18	0.0000
Local governments/municipal corporations should have stray cat sterilizing programs in the cities/towns to control their population	SD = 63, 8.26%	Gender	11.01	0.2013
	D = 99, 12.98%	Age	11.21	0.5111
	N = 93, 12.19%	Education	40.68	0.0006
	A = 242, 31.72%	Own-pet	139.34	0.0000
	SA = 262, 34.34%	Feeder (y/n)	65.68	0.0000

For the second item, “stray cat numbers should be reduced by sterilizing them so they are unable to have more kittens,” 64.9% agreed, 21.1% disagreed, and 13.9% were unsure (Table 4). Similarly, when asked whether “local governments/municipal corporations should have stray cat sterilizing programs in the cities/towns to control their population” 66.1% agreed, 21.2% disagreed, and 12.2% were unsure. For both these items there was no difference across genders, age groups or education level.

Pet- and cat-owners less likely to agree with any form of stray cat population control and significantly more opposed to euthanasia. Stray cat feeders were also significantly more opposed to managing stray cat population than those who had never fed them.

## 4. Discussion

A city is home not only to humans with their interests, but is an environment of cohabitation with animals. Despite the fact that animals live in the built environment, urban planning and initiatives have almost exclusively been approached from an anthropocentric lens, focusing on human health and happiness (41–44). Nonetheless, animals exist in urban areas, and need to be considered in contemporary and future urban environments, particularly in countries with rapidly changing urban landscapes. With the One Health approach, we recognize that the health of people and cats is connected, with risk of zoonotic disease transmission (45).

Furthermore, humans are causally responsible for the presence of animals in urban areas, having taken over natural habitats of animals and introducing animals into urban areas (41). Ethicists have argued that, at a minimum, animals must not be condemned for their presence in urban spaces (41). Urban stray cats living within human social constructs are directly or indirectly dependent on humans for food. They scavenge in garbage, hunt mice and rats in human settlements, or find humans who will feed them. As such, overpopulation of stray cats in an urban area is an anthropogenic problem, one that humans are responsible for resolving (46).

This study was the first to investigate attitudes towards stray cats and population control in India. The high rate of stray cat sightings reported aligns with a high estimated number of stray cats in India, with recent reports estimating a stray cat population of 9.1 million (22). With a large population of stray cats in a dense urban setting, a great degree of interaction with humans is expected. 73.3% of our respondents had some form of interaction, whether this included shooing off cats, kicking/hitting them, being bitten/scratched by them, feeding them, taking them to the vet, or adopting them.

Internationally, a wide range of attitudes towards stray cats is reported. In Brisbane, Australia, participants believed stray cats exhibit nuisance behaviors (34). In Japan, over a third of respondents complained about urine and feces from cat colonies (29), whereas in Bulgaria (19) participants held positive attitudes towards stray cats and did not consider them to be a nuisance. In our study, participants were observed to have varying attitudes towards stray cats. Nearly two of three participants agreed that the welfare of stray cats is important, yet most participants had negative attitudes towards them. Over 60% agreed that cats are “dirty and stink up the place where they are” and “are a nuisance and disturb humans with their loud fighting.” Further, one in two people who agreed with both these items had shooed off a stray cat.

Over 60% of respondents in our study believed that stray cats spread diseases to humans. Elsewhere, studies found that one in five respondents believe stray cats spread disease to humans (34). However, it has been shown that cats pose a low disease transmission-risk to humans, and it is even lower from stray cats (47). Public education campaigns can play an important role in providing evidence-based information and dispelling unfounded beliefs.

Women in our study were more likely to have shooed off stray cats. This does not align with previous studies reporting women exhibiting greater positive attitudes towards animals (48), and less likely to perceive cats’ nuisance factors (49). Our findings may result from Indian women shouldering the burden of domestic work. Indian women are primarily tasked with household chores including cooking and cleaning (50, 51). There may be greater interaction between women and stray cats who forage in household garbage, have litters in corners of low-income houses removed from the street’s dangers, and other instances of cats breaching porous boundaries between the street and home, and therefore, be subject to being shooed off.

Younger respondents (aged <30 years) in our study were less likely to hold negative views towards stray cats or have shooed them off. They were also significantly more likely to have taken a stray cat to a vet. This aligns with previous studies reporting younger participants as more empathetic towards animals (52, 53). Since 52% of India’s population is under 30 years of age, targeted campaigns to augment and retain this positive attitude in the younger residents has the potential for a long-term improvement in the welfare of stray cats in India (54).

Our finding of a more positive attitude in the younger population may also be related to the global evolution in the relationship between humans and cats. Historically, the primary approach to stray cat population control was to euthanize cats in large numbers (55). In the past three decades there has been an ethical shift away from euthanasia (10, 56). We found a low level of support for euthanasia to control stray cat populations, with younger respondents more opposed. The overall low level of support for euthanasia in our respondents may also be reflective of Indian cultural values associated with animal life. We did not collect information on religion, but there may be a religious element to opposing euthanasia, given that Jainism and Buddhism, two common religions in India, oblige followers to protect all forms of life (57–60). Further, it is against Indian law to kill stray animals (61, 62). With recent incidents of attack between citizens and stray dogs, there has been considerable news media attention to the welfare of stray animals in India (63–65). Our respondents may have been aware of the legal code prohibiting killing of stray animals.

Globally, TNR programs are increasingly employed as a humane, effective approach to managing urban stray cat populations (10, 13, 15). TNR is the method of choice in the International Society of Feline Medicine’s Welfare Advisory Panel (56). In 2018, the AWBI advised states and municipal authorities to undertake spay/neuter activities for population control for stray cats, but there is no broad effort towards TNR programs for stray cats in India. In Mumbai, a nascent stray cat TNR program is modelled on the long-standing and successful Animal Birth Control program for stray dogs. However, unlike dogs, cats are far more challenging to identify and catch. In order to effectively implement TNR for stray cats, public support is essential. Our study found a widespread lack of awareness about TNR programs, with nearly three out of four participants never having heard of TNR, even when described in lay language. However, when presented with a description of TNR, the respondents were supportive, and TNR programs implemented by local governments would potentially be well-supported by urban Indian residents. Further, given concerns about odors and noises, TNR’s effectiveness to reduce these nuisance behaviors could be stressed in education campaigns to build community support.

Urban stray cats are often fed and cared for by voluntary “cat-feeders.” Caring for stray cat colonies, including feeding, is important in trapping cats as part of TNR (66). In our study, 36.7% of respondents reported feeding stray cats. Studies in Australia and US report 9–26% feeding rates (16, 26, 35, 37). Feeders in our study were across both genders, young and old, with or without college education with no statistically significant difference. Studies show the average feeder is in middle adulthood (26, 30, 38, 67, 68), yet people of all ages feed cats (39, 69). Reasons for feeding include empathy, affection, compassion, a sense of responsibility for hungry animals, and a nurturing need (30, 38, 67, 69). Furthermore, feeders believe they are helping improve the cats’ welfare (30, 70). This is often a point of contention between those who care for the strays and others who perceive them as a nuisance (30). In our study, when asked whether people who are feeding stray cats were creating a bigger problem, older people and those without college education tended to agree. They also disagreed with the statement that “feeding a stray cat would make me feel good.” Elsewhere, attitudes towards stray cats have been shaped by communications interventions to increase the acceptance for TNR programs (24). As such, public education on the importance of feeding stray cats towards stabilization of their population needs to be considered as part of the TNR program rollout in India.

Positive attitudes towards stray cats are also associated with pet- and cat-ownership (71). This was consistently borne out in our study. Pet ownership has been correlated with higher empathy (72–74). This was supported by our findings that pet- and cat-owners were significantly more likely to disagree with euthanasia. While there is limited insight into pet ownership in India, our study found that 36.4% of respondents currently/ever owned a pet, including 22.7% having ever owned a cat. Industry estimates there were 32 million pets in India in 2022, over double the estimate from 2017, with the younger population fueling pet-ownership (75). Public education to encourage and empower residents to adopt stray cats who are deemed suitable for adoption will not only positively impact the lives of the adopted strays, but could result in increased sensitivity towards, and improved welfare of, cats across India.

### 4.1. Limitations

There are limitations to our study, in the methods and in interpreting our findings. Limitations in the methods include lack of back translation of the surveys and that the reliability of the questions within the questionnaire was not measured. In interpreting the findings, broadly, quantitative analyses of surveys to study human-animal relationships have been questioned methodologically as unable to capture complex and nuanced factors (1). As such, this is a potential limitation. However, given that this is a pilot study, and the first study in India, we believe the survey can be helpful in understanding baseline information and inform further studies and initiatives to ultimately improve the welfare of stray cats in India. More specifically to our study, in a country as diverse as India, it is not anticipated that our sample is representative of the varied populace. Although the age distribution was close to the actual age distribution, younger people were slightly under-represented. Our sample was more educated than the profile in Indian cities. Further, based on estimates of pet ownership in India, our sample had a higher proportion, and may have resulted in a lower level of negativity towards stray cats than the general population. Religious and economic indicators, potentially closely associated with attitudes towards cats, are missing. As such, this is a limitation of our study.

## 5. Conclusion

The study was an exploratory, pilot study, as a first examination of the issue in India. We hope the results from our study will prompt researchers to investigate these attitudes in depth, thereby allowing policymakers, urban planners and animal welfare advocates to reflect upon, and systematically plan, a sustainable future for the cats who share our cities with us. As low and lower-middle income countries are entering an urban age, we have a moral obligation to create sustainable communities that include not just humans but also the rest of the natural world. Albeit TNR has not been deployed at a broad scale, or studied in depth, in a densely populated, urban setting with resource challenges similar to India, it currently presents as a potential option to curbing the population of stray cats while also improving their welfare. Importantly, using public health education interventions to shape positive attitudes towards the millions of animals who share our streets could lead to a socio-cultural shift in how they are viewed: as integral members of our cities that they are.

## Data availability statement

The raw data supporting the conclusions of this article will be made available by the authors, without undue reservation.

## Ethics statement

Ethical approval was not required for the studies involving humans because this non-interventional involved a survey. Participants were informed of full anonymity with no identifiable data being collected, explained why the research is being conducted, how their data would be used and of any risks to obtain informed consent before participating. Ethical approval according to local and national laws was not needed for this type of study. The studies were conducted in accordance with the local legislation and institutional requirements. Written informed consent for participation was not required from the participants or the participants’ legal guardians/next of kin in accordance with the national legislation and institutional requirements because as per the national ethical guidelines, it is eligible for a waiver of written informed consent, as the study involves less than minimal risk to participants, participants are de-identified and cannot be contacted, and the waiver does not adversely affect the rights and welfare of the participants.

## Author contributions

AC-R: Conceptualization, Methodology, Writing – original draft. NT: Formal analysis, Writing – review & editing.
