# Supplementary material for: Attitudes towards urban stray cats and managing their population in India: a pilot study
Source: Front Vet Sci. 2023 Oct 27;10:1274243. doi: 10.3389/fvets.2023.1274243 (PMC10641441; doi:10.3389/fvets.2023.1274243)
Supplement: Supplementary file 1 [file Data_Sheet_1.pdf]

Stray Cats In India

Hello, I’m working on a study to collect information on stray cats in India. As you may be aware, the expansion of cities has had a big impact on stray animals. Your feedback will help in the management of stray cats. The questions will take about 5-7 minutes to answer. The survey is anonymous. Please answer each question as honestly as possible, as this will help in getting a good picture of the real situation. Thank you!

For the following questions, please answer Yes or No:

1. Are you aware of any stray cats in your neighborhood where you live or around where you work/study?

   Yes No

2. Have you seen a stray cat in the past month?

   Yes No

3. Have you ever heard about programs to catch, sterilize (so they can’t have any kittens) and return stray cats to their neighborhood?

   Yes No

4. Have you ever shooed off a stray cat who you thought was being a nuisance?

   Yes No

5. Have you ever been bitten or scratched by a stray cat?

   Yes No

6. Do you feed or have you ever fed any stray cats?

   Yes No

7. Have you ever taken a stray cat to a vet?

   Yes No

8. Have you ever adopted a stray cat and taken it home?

   Yes No

For the following questions, please choose your response from:
Strongly Agree, Somewhat Agree, Unsure, Somewhat Disagree, Strongly Disagree

	Question	Strongly Agree	Somewhat Agree	Unsure	Somewhat Disagree	Strongly Disagree
9	The welfare of stray cats is important					
10	Stray cats are dirty and stink up the place where they are					
11	Stray cats spread diseases to humans					
12	Stray cats are a nuisance and disturb humans with their loud fighting					
13	People who feed stray cats are creating a bigger problem					
14	Local governments/municipal corporations should remove all the stray cats and euthanise (kill) them like they used to do with dogs					
15	Stray cat numbers should be reduced by sterilizing them so they are unable to have more kittens					
16	People who feed stray cats are improving the welfare of cats					
17	Feeding a stray cat would make me feel good					
18	Local governments/municipal corporations should have stray cat sterilizing programs in the city to control their population					

The next few questions are about you:

19. Have you ever had a pet at home (currently or in the past)? Please check all that apply.

Never had any pets Cat/s Dog/s Other pet/s

20. Gender

Male Female Other Would rather not say

21. Age

18-29 30-44 45-59 60 and over

22. Education

No formal qualifications

Primary school up to 4th standard

Secondary school up to 10th standard

Some college, but not graduated

College graduate or higher

23. City/Town of Residence

Ahmedabad

Bengaluru

Bhubaneshwar

Chennai

Delhi

Kochi

Kolkata

Lucknow

Mumbai

Nagpur

Patna

Varanasi

Vijayawada
